# Study of Brain Wounds

**Published:** 1918-08

**Authors:** 


**Study of Brain Wounds.** By Lt.-Colonel HARVEY CUSHING, M. R. C., *British Journal of Surgery*, April 19, 1918.

Between July 23 and Oct. 31, 1917, Cushing studied 219 injuries of this character. 22, or 10 o/o, were scalp wounds; 54 or 24.6 o/o were scalp and cranium wounds with intact dura; 133 or 60.7 o/o were scalp, cranium, and dura wounds; and 10, or 4.5 o/o, were scalp and cranium wounds with bursting fractures. In addition there were 20 cases upon whom no operation was possible on account of the bad condition and 11 upon whom no operation was deemed advisable. Of the 133 complicated cases, in the last 45 the mortality dropped from 54 o/o to 28.8 o/o — the technique employed being block removal of bone involved, with excision as done in ordinary wounds. The wound in the brain was washed out with saline solution introduced with syringe and soft catheter. By alternate washing out and suction — all under local anesthesia — the results have improved markedly. The mortality in this series was as follows :

1. Wounds of the scalp with intact cranium and dura : 22 cases in all; 1 death.

2. Wounds of the scalp, local fracture with or without depression, with contusion of brain, dura intact : 54 cases in all; 5 deaths.

3. Local depressed fracture with punctured dura usually with neurological symptoms, but not accompanied by cerebral extrusion : 18 cases; 2 deaths.

4. Wounds usually of gutter type with detached fragments driven into brain with severe cerebral contusion and extrusion of brain, leading frequently to fungus and encephalitis : 25 cases in all; 6 deaths.

5. Wounds of penetrating type with lodgment both of projectile and bone fragments, the brain often extruding, marked contusion along tract, leading frequently to early compression or late abscess : 41 cases in all; 15 deaths.

6. Penetrating or traversing ventricles either by projectile or bone fragments, brain protusion likely or common, hemorrhage or infection of ventricles frequent with escape of cerebro-spinal fluid, when the ventricles were penetrated or traversed by fragments of bone : 14 cases in all; 6 deaths.

7. If penetrated or traversed by projectiles : 16 cases in all; 16 deaths.

8. Wounds with involvement of the orbital nasal or auro-petrosal areas frequently leading to meningitis : 15 cases in all; 11 deaths.

9. Cranial cerebral through and through : 5 cases in all; 4 deaths.

10. Wounds with massive comminution of skull : 10 cases in all; 5 deaths.

Cushing says : " We may say that unless it is proved by investigation a fracture may be supposed to underly every scalp wound made by a projectile " (page 568). " There is no justification in withholding operation from those unfortunates for whom a prolongation of life would seem undesirable... unexpected recovery with unimpaired mental faculties sometimes following what appear to be the most extensive cerebral injuries. "

In studies of 1239 cases by Sargent and Holmes and of 6664 cases by Tuffier and Guillan, recoveries after trephining without epilepsy, insanity, abscess, crippling paralysis, etc., are much more common than would have been anticipated.

The surgical treatment of Group 2, if the external table is depressed, should be block trephining, cleaning out all damaged tissues. If the external table is not broken, individual judgment will decide usually from symptoms as to whether the internal table should be exposed. In groups 3, 4, 5, 6, 7, 8 and 9 — block trephine must be done whenever possible, with careful débridement of all bruised or dead tissues. Care must be taken not to open up the dura widely because of the danger of spreading infection. In fact Cushing attempts without enlarging the dural opening to clean out the damaged cerebral tissue. In Group 10 — a subtemporal decompression is indicated.

In injuries on the top of the head, attention is called to the longitudinal sinus syndrome which is due to a contusion of the mesial aspect of the hemispheres and is evidenced by bilateral palsies, exaggerated deep reflexes, and Babinski. In these cases Sargent and Holmes have advised against trepanation. After the operation or following trauma, repeated Jacksonian attacks in the early stages may develop, but they usually subside. In controlling bleeding from the sinuses Cushing reports satisfactory results with muscle graft. In the treatment of fungus cerebri and in washing out brain tracts which are drained, Cushing uses Dichloramine T. He has been able at times to wash out or suck out fragments of bone located in the ventricles. Magnet extraction of metal bodies has worked well. When there are large defects in the scalp, Cushing advises against primary plastic closure, except when the ventricle has been opened. Whenever the dura can be closed to avoid leakage and infection, he advises that this be done. Studies of the optic disc have shown edema more frequently than would have been expected especially after the severe injuries. In one case distinct gas encephalitis caused by the bacillus perfringens was observed.

